# *In vitro* study on synergistic antifungal effects of docetaxel combined with azoles against dematiaceous fungi and *Aspergillus niger*

**DOI:** 10.3389/fmicb.2026.1832946

**Published:** 2026-07-17

**Authors:** Heng Zhang, Hua Ni, Mengqi Peng, Tian Chen, Lu Ge, Xueying Zhang, Kai Tian, Yi Sun

**Affiliations:** 1 Department of Dermatology, Hubei Provincial Clinical Research Center for Diagnosis and Therapeutics of Pathogenic Fungal Infection, Jingzhou Hospital Affiliated to Yangtze University, Jingzhou, Hubei, China; 2 Department of Nephrology, Hubei Provincial Clinical Research Center for Diagnosis and Therapeutics of Pathogenic Fungal Infection, Jingzhou Hospital Affiliated to Yangtze University, Jingzhou, Hubei, China; 3 Department of Clinical Medicine, Yangtze University, Jingzhou, Hubei, China

**Keywords:** antifungal activity, *Aspergillus niger*, azoles, dematiaceous fungi, docetaxel, *Exophiala dermatitidis*, synergism

## Abstract

**Background:**

Dematiaceous fungal infections are frequently encountered in clinical practice with no unified treatment. Drug combination reduces drug resistance, and repurposed clinical drugs as synergists show promise in aiding azoles against phaeohyphomycosis.

**Objective:**

To investigate the synergistic antifungal effects of docetaxel (DTX) in combination with azoles against dematiaceous fungi and *Aspergillus niger*, as well as their impact on efflux pump activity.

**Methods:**

CLSI M38-A2 microbroth dilution method was used to detect fractional inhibitory concentration index of DTX with posaconazole (POS), itraconazole (ITC), voriconazole (VOR), and isavuconazole (ISA) against 43 fungi (including 24 strains of *Exophiala dermatitidis*, 7 strains of *Exophiala alcalophila*, 3 strains of *Fonsecaea pedrosoi*, 1 strain of *Fonsecaea monophora*, 1 strain of *Exophiala verrucosa* and 7 strains of *Aspergillus niger*). Rhodamine 6G transport efficiency and transcriptional variations of efflux pump genes were determined in strains treated with POS alone or POS combined with DTX.

**Results:**

DTX alone had no antifungal activity. 30/43 (69.8%) strains showed synergy with POS (87.5% *E. dermatitidis*, 100% *A. niger*). 7 (16.3%), 3 (7.0%) and 1 (2.3%) strains showed synergy with ITC, VOR and ISA. For strains with POS synergism, DTX treatment significantly suppressed efflux activity and modified the transcription of efflux pump genes.

**Conclusion:**

The combined scheme of DTX and POS can significantly reduce the minimum inhibitory concentration of POS and show synergistic antifungal activity, especially significant against *E. dermatitidis* and *A. niger*. Its mechanism of action may be related to the reduction of efflux pump activity.

## 1 Introduction

Fungi containing dark pigments (melanin) in their cell walls are referred to as dematiaceous fungi, which include genera such as *Exophiala*, *Fonsecaea*, and *Phialophora* (Revankar, 2006; Wong et al., 2016). *Aspergillus niger*, though also a melanin-producing fungus, does not belong to the dematiaceous fungi group taxonomically. These melanized fungi can cause phaeohyphomycosis, which is particularly prevalent in immunocompromised patients (Cavens et al., 2025). For example, the black yeast *Exophiala dermatitidis* (phylum Ascomycota, order Chaetothyriales) is renowned for its ability to thrive in diverse harsh environmental conditions and exhibits polyextremotolerance to various stressors (Cavens et al., 2025; Kirchhoff et al., 2019; Teixeira et al., 2017). It is an opportunistic pathogen that can cause global infection epidemics, leading to a range of diseases including skin infections, suppurative arthritis, endocarditis, catheter-related fungemia, and systemic infections, with a 40% mortality rate (Cavens et al., 2025; Kirchhoff et al., 2019; Poyntner et al., 2016,^2018^; Teixeira et al., 2017).

Dematiaceous fungi can also induce chromoblastomycosis, an implanted mycosis acquired through traumatic inoculation, clinically characterized by chronic granulomatous infections of the skin and subcutaneous tissues. This disfiguring fungal infection was included in the World Health Organization’s list of neglected tropical diseases in 2017, among which *Fonsecaea pedrosoi* is the most common pathogen worldwide (Queiroz-Telles et al., 2017; Smith et al., 2025; Smith et al., 2024). In addition, *A. niger* frequently serves as an etiologic agent of otomycosis and can be highly invasive in immunocompromised patients, acting as an opportunistic pathogen that often results in high mortality, especially in lung transplant recipients (Bojanovic et al., 2023; Rafique et al., 2026).

Currently, there are no global treatment guidelines for chromoblastomycosis; however, prolonged therapy and surgical intervention are typically required, with treatment courses lasting from several months to years. The mainstay of current treatment still relies on classic azole drugs (Robbins et al., 2017). Nevertheless, the increasing incidence of azole resistance has hampered therapeutic efforts. Key mechanisms contributing to azole resistance include the overexpression of drug efflux pumps (such as ATP-dependent ABC transporters and MFS transporters), as well as mutations or increased expression of the CYP51 gene (*ERG11* in yeast) that encodes 14-*α*-demethylase (Perez-Cantero et al., 2020; Robbins et al., 2017).

**Figure 1:**
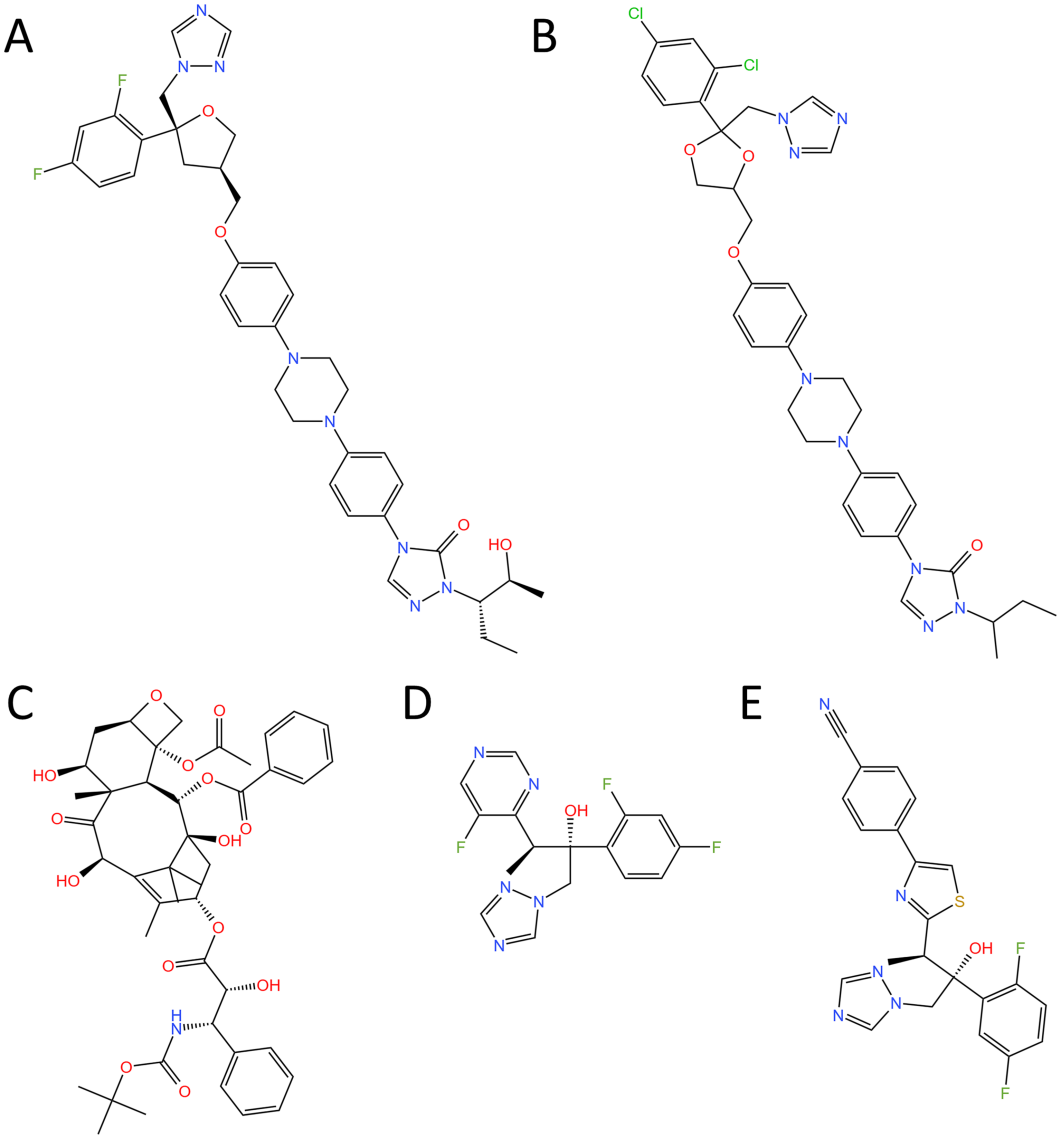
Chemical structures of posaconazole, itraconazole, docetaxel, voriconazole, and isavuconazole. Schematic chemical structures of the tested antifungal and chemotherapeutic agents. **(A)** Posaconazole; **(B)** Itraconazole; **(C)** Docetaxel; **(D)** Voriconazole; **(E)** Isavuconazole.

To address these challenges, combined antifungal therapy has emerged as a promising strategy. Docetaxel (DTX) is a semi-synthetic taxane drug obtained by extracting a natural taxane precursor (10-deacetylbaccatin III, 10-DAB) from *Taxus baccata* (European yew) followed by artificial chemical modification (Figure 1; Harada et al., 2019; Hirata et al., 2024). This transformation from a natural plant component to a clinical chemotherapeutic drug not only retains the core pharmacological scaffold of taxanes but also enhances antitumor activity and clinical applicability through structural optimization, making it one of the most effective drugs in chemotherapy. The mechanism by which this drug acts on cancer cells involves inhibiting microtubule depolymerization, while its ultimate elimination *in vivo* is mediated by the P-glycoprotein (P-gp) efflux pump in the intestine (Horwitz et al., 2025; Petrylak et al., 2004; Saremi et al., 2011). *β*-tubulin is the shared target of DTX and paclitaxel, and stabilization of microtubules by these agents leads to cell cycle arrest and apoptosis (Bernkop-Schnurch et al., 2001; Pienta, 2001; Saremi et al., 2011). Initially, cancer cells are sensitive to paclitaxel treatment, but over time, paclitaxel resistance develops through multiple mechanisms. Resistance of cancer cells to paclitaxel arises from the activation of drug efflux proteins, alteration of molecular pathways related to apoptosis, and upregulation of the expression of taxol resistance-associated gene 3 (*TRAG-3*/*CSAG2*). For instance, DTX resistance has been observed in prostate cancer (PCa) cells, with known mechanisms including limited intracellular drug concentration. Overexpression of several ABC transporters has been identified as a cause of chemotherapeutic drug efflux, and the development of agents capable of regulating efflux pump-mediated multidrug resistance (MDR) remains a key goal in the treatment of PCa (Demidenko et al., 2015; Hwang, 2012). This also suggests that DTX may exert potential antifungal effects by interfering with the activity of fungal efflux pumps. Previous studies have confirmed that itraconazole (ITC) can bind to human ATP-binding cassette (ABC) transporter ABCB1 and reverse ABCB1-mediated DTX resistance in PCa (Lima et al., 2022). This indicates that the combination of azoles and DTX has the potential to modulate the activity of ABC transporters. By analogy, azoles may also target fungal ABC transporters; when combined with DTX, they can alter the process of antifungal efflux, thereby regulating fungal susceptibility to azole agents. In this study, the microdilution method was used to evaluate the synergistic effects of DTX in combination with posaconazole (POS), ITC, voriconazole (VOR), and isavuconazole (ISA), respectively, against dematiaceous fungi.

## 2 Materials and methods

### 2.1 Fungal strains, antifungals, and chemical agents

In this study, 43 clinically isolated strains were included (Supplementary Table S1) (Jia et al., 2023). All the strains were reconfirmed by performing microscopic morphology and molecular identification techniques. Fungal DNA was extracted, and the internal transcribed spacer (ITS) region of ribosomal DNA (rDNA) was sequenced for fungal identification (Supplementary Table S2). Susceptibility testing was also performed with *Candida parapsilosis* ATCC22019 and *Aspergillus flavus* ATCC204304 as control strains to ensure quality control.

All strains used in this study were stored at −80 °*C. prior* to experiments, strains were subcultured twice on Sabouraud Dextrose Agar (SDA, Haibo Biological) at 35 °C. *A. niger* was incubated for 3 days, whereas the other strains were cultured for 7 days. Fresh conidia were harvested with sterile distilled water containing 0.03% Triton X-100 and quantified under a microscope using a hemocytometer. Subsequently, susceptibility testing was performed to ensure the viability and purity of the tested fungi.

The experimental drugs, such as ITC (Cat. No. HY-17514, purity = 99.59%), POS (Cat. No. HY-17373, purity = 99.95%), and VOR (Cat. No. HY-76200, purity = 99.45%), were obtained from MedChemExpress (MCE), NJ, USA. DTX (Cat. No. D107319, purity ≥ 99%) and ISA (Cat. No. I337027, purity ≥ 98%) were purchased from Aladdin Biotech Co., Ltd., China.

All drugs were solubilized in dimethyl sulfoxide (DMSO), with the stock solution concentration standardized at 6400 μg/mL.

### 2.2 Strain identification

The cultured fungal specimens were taken and preliminarily identified according to the morphological characteristics. Fungal DNA was extracted using the MolPure Fungal DNA Kit (Yeasen Biotech, Shanghai, China). The ITS region of ribosomal DNA (rDNA), beta-tubulin, and calmodulin genes were further amplified (Supplementary Table S2) (Zhang et al., 2025). PCR was performed using the following parameters: 3 min at 95 °C, followed by 35 steps of 1 min at 95 °C, 1 min at 58.5 °C and 1 min at 72 °C, and then a final 10 min at 72 °C. The final products were sequenced by Biocompany [BioEngineering (Shanghai) Co., Ltd], and finally, the sequence was blasted in NCBI GenBank. The definitive identification of the isolates was accomplished by comparing the sequences with relevant reference sequences in GenBank using the nucleotide BLAST system.^1^ These sequence data have been submitted to the GenBank databases under accession number PP069948–PP070390.

### 2.3 *In vitro* combined drug sensitivity

The *in vitro* interactions between DTX and azoles against pathogenic fungi were tested via the microdilution chequerboard technique, adapted from the Clinical and Laboratory Standards Institute (CLSI) broth microdilution method M38-A2 (Zhang et al., 2025). The conidial suspension was adjusted to a concentration of 1 × 10^6^ to 5 × 10^6^ CFU/mL, followed by a 100-fold dilution with RPMI 1640 medium. The resulting suspension was twice the target working concentration for subsequent experiments, corresponding to approximately 1 × 10^4^ to 5 × 10^4^ CFU/mL. Serial diluents of tested agents were prepared as outlined in the M38-A2 protocol by dilution with RPMI 1640. The working concentration ranges were 0.0625 to 8 μg/mL for ITC, VOR, and ISA, 0.03125 – 4 μg/mL for POS, and 1 to 64 μg/mL for DTX. As described, 50 μL of DTX with serial dilutions was inoculated in horizontal direction and another 50 μL of azoles with serial dilutions was inoculated in a vertical direction on the 96-well plate, which contained 100-μL prepared inoculum suspensions. *A. niger* cultures were incubated at 35 °C for 2 days, while all other strains were cultured for 3 days. The minimum inhibitory concentration (MIC) was defined as the lowest concentration that completely inhibits visible fungal growth, with MIC values determined for each drug in both single-drug and combination regions. All susceptibility tests were performed in triplicate. The fractional inhibitory concentration index (FICI) was calculated as the sum of the MIC of each drug in combination divided by its MIC alone: FICI = (MIC of A in combination/MIC of A alone) + (MIC of B in combination/MIC of B alone). Interactions were categorized as follows: synergistic (FICI ≤ 0.5), no interaction (0.5 < FICI ≤ 4), or antagonistic (FICI > 4) (Jia et al., 2023). The maximum concentration of DMSO in all wells was less than 1%.

### 2.4 Rhodamine 6G efflux assay

The intact cell efflux assay of rhodamine 6G (R6G) was modified based on the method described by Kolaczkowski et al. (1996). Conidia from SDA cultures in the exponential growth phase (OD600, 0.5) were collected by centrifugation (3,000 × g, 5 min, 20 °C) and washed three times with water. The washed conidia were equally allocated into four treatment groups, namely a solvent control 1 supplemented with an equal volume of DMSO matching the solvent amount for POS monotherapy (no antifungals), a solvent control 2 supplemented with an equal volume of DMSO matching the total solvent volume of DTX + POS combination (no antifungals), a POS monotherapy group incubated with RPMI 1640 medium containing POS at half its single-drug MIC, and a DTX + POS combination group cultured in RPMI 1640 medium with POS and DTX co-administered, with each drug adjusted to half of their respective combinatorial MIC measured in the DTX + POS combination regimen. Both groups were incubated with shaking (200 rpm) at 35 °C for 16 h, followed by three washes with PBS (pH 7.2) to completely remove residual drugs. Subsequently, the treated cells from both groups were resuspended separately in HEPES-NaOH buffer (50 mM, pH 7.0) containing 5 mM 2-deoxyglucose and 10 μM R6G, adjusted to a concentration of 0.5 × 10^6^–1.0 × 10^7^ CFU/mL, and incubated with shaking (200 rpm) at 30 °C for 90 min to allow R6G accumulation in cells under glucose starvation conditions. After that, the starved cells were washed twice with HEPES-NaOH buffer; 400 μL of the cell suspension was incubated at 30 °C for 5 min, and then 2 mM glucose was added to initiate the R6G efflux process. At specific time points (5, 10, 15, 20, and 30 min) after glucose addition, cells were removed by centrifugation, and 100 μL of cell supernatant (three replicates set for each time point) was transferred to a 96-well flat-bottom microtiter plate (BKMAM Biotechnology, Hunan, China). The rhodamine fluorescence of the cell supernatants was measured using a microplate reader (ALLSHENG, Wuhan, China) at an excitation wavelength of 529 nm and an emission wavelength of 553 nm.

### 2.5 Quantification of gene expression by RT-qPCR

Fresh conidia of *E. dermatitidis* and *A. niger* were inoculated into liquid SDA medium at a final concentration of 2 × 10^7^ CFU/mL, followed by shaking incubation at 37 °C and 200 rpm for 72 h and 36 h, respectively. POS (at half of its single-drug MIC) or the DTX + POS combination (each agent at half of the combined MIC) was supplemented into the cultures, and incubation continued for an additional 12 h prior to three washes of the cell pellets. Total RNA was extracted with theTRIeasy™ Total RNA Extraction Reagent (Yeasen, China) according to the manufacturer’s directions. The Hifair® III 1st Strand cDNA Synthesis SuperMix for qPCR (Yeasen, China) was used to synthesize cDNA. Independent assays were performed with three replicates, and transcript levels were calculated by the comparative threshold cycle (ΔCT) and normalized against the mRNA expression of *actin*. The 2^−ΔΔCT^ method was used to determine the changes in mRNA expression (Livak et al., 2001).

### 2.6 Statistical analysis

GraphPad Prism 9 was used for plotting graphs, and SPSS 26.0 was applied for all statistical analyses. All experiments in this study were performed with three biological replicates, and each biological replicate contained three technical replicates. All data were expressed as mean ± standard deviation (SD). Intergroup differences were assessed via two-way analysis of variance (two-way ANOVA) with Tukey’s *post hoc* test. *p* < 0.05 was considered statistically significant.

## 3 Results

### 3.1 *In vitro* interactions between DTX and azoles against dematiaceous spp

When used alone, DTX exhibited no antifungal activity against dematiaceous fungi. In the drug combination system, the MIC of POS alone against dematiaceous fungi ranged from 0.0625 to 4 μg/mL; however, after the combined use of DTX and POS, the MICs of both drugs decreased significantly: the MIC of DTX in combination was 2–16 μg/mL, representing a 32-fold reduction from its highest tested single concentration (64 μg/mL), and the MIC of POS decreased to 0.03125–2 μg/mL (also with a maximum reduction of 32-fold).

**Figure 2:**
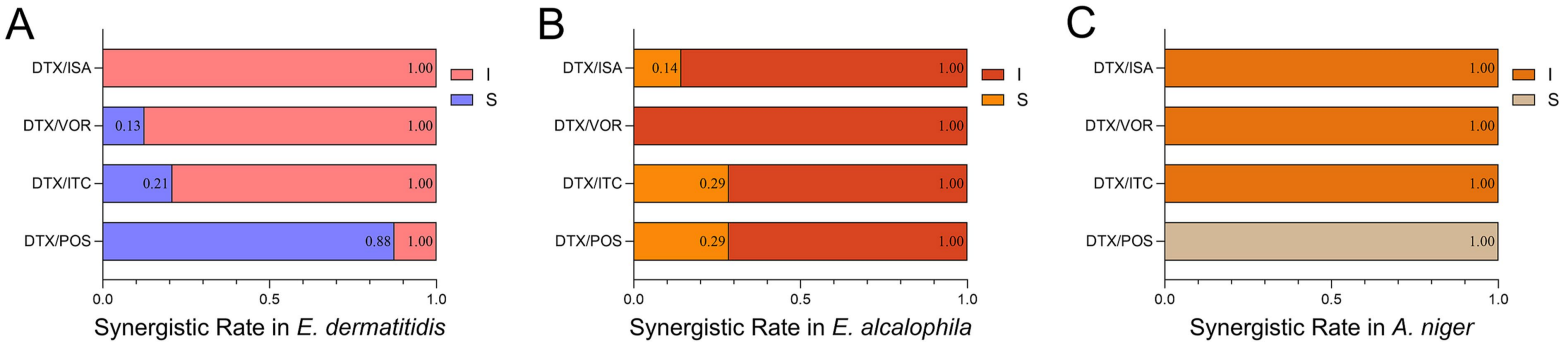
The synergistic rate of DTX combined with azoles. **(A)** Interaction profile of DTX combined with POS, ITC, VOR, and ISA against *E. dermatitidis*; **(B)** interaction profile of DTX combined with POS, ITC, VOR, and ISA against *E. alcalophila*; **(C)** interaction profile of DTX combined with POS, ITC, VOR and ISA against *A. niger*. The synergism rate was calculated by dividing the number of strains exhibiting synergism by the total number of strains tested. S: synergy (FICI≤0.5); I: indifference (FICI from >0.5 to ≤4); A, antagonism (FICI of >4). DTX, docetaxel; ITR, itraconazole; VOR, voriconazole; POS, posaconazole; ISA, isavuconazole.

A detailed analysis of the synergistic effects on clinical isolates of different fungal species showed the following results: Among 24 strains of *E. dermatitidis*, 21 strains exhibited a synergistic effect with the DTX + POS combination, 5 strains showed synergy with the DTX + ITC combination, and 2 strains displayed synergy with the DTX + VOR combination. However, no synergistic effect was observed between any of these strains and the DTX + ISA combination (Figure 2A). Among the 7 strains of *E. alcalophila*, 2 showed synergy with the DTX + POS combination, 2 with the DTX + ITC combination, and 1 with the DTX + ISA combination (Figure 2B). For the 7 strains of *A. niger*, all strains produced a synergistic effect with the DTX + POS combination, while no synergy was detected in the DTX + ITC, DTX + VOR, or DTX + ISA combinations (Figure 2C). In addition, no synergistic effects between DTX and any azole were detected in the 3 strains of *F. pedrosoi*, 1 strain of *F. monophora* or 1 strain of *E. verrucosa* (Table 1).

**Table 1:** *In vitro* drug sensitivity results of DTX combined with azoles against dematiaceous strains.

Strains	MIC alone (μg/mL)					MIC combinations (μg/mL)			
	DTX	POS	ITC	VOR	ISA	DTX/POS	DTX/ITC	DTX/VOR	DTX/ISA
*E. dermatitidis*									
BMU00028	>64	1	1	0.5	2	16/0.25(S)	16/0.25(S)	64/0.5(I)	1/1(I)
BMU00029	>64	1	1	1	1	16/0.25(S)	64/2(I)	8/0.25(S)	64/2(I)
BMU00030	>64	1	1	1	2	8/0.5(I)	4/0.5(I)	4/0.5(I)	1/1(I)
BMU00031	>64	1	1	1	2	4/0.25(S)	64/2(I)	4/0.5(I)	64/4(I)
BMU00034	>64	1	2	1	2	4/0.25(S)	16/0.5(S)	8/0.25(S)	64/4(I)
BMU00035	>64	0.5	1	0.5	2	16/0.125(S)	16/0.25(S)	64/1(I)	64/4(I)
BMU00036	>64	0.5	1	0.25	0.5	2/0.125(S)	4/0.25(S)	1/0.125(I)	64/1(I)
BMU00037	>64	1	1	0.25	2	2/0.25(S)	64/2(I)	64/0.5(I)	64/4(I)
BMU00038	>64	0.5	1	0.5	4	4/0.125(S)	4/0.5(I)	64/0.5(I)	64/8(I)
BMU00039	>64	1	1	0.25	1	16/0.25(S)	64/2(I)	64/0.5(I)	64/2(I)
BMU00040	>64	0.25	0.5	0.125	0.5	2/0.0625(S)	32/0.25(I)	64/0.25(I)	64/1(I)
BMU00041	>64	0.5	1	0.25	1	2/0.125(S)	64/2(I)	64/0.5(I)	64/2(I)
109140	>64	1	1	0.5	2	64/2(I)	64/2(I)	64/1(I)	64/4(I)
109144	>64	1	1	1	0.5	8/0.25(S)	64/1(I)	64/0.5(I)	64/0.25(I)
109145	>64	1	1	0.5	2	16/0.25(S)	16/0.25(S)	64/1(I)	64/4(I)
109148	>64	1	2	0.5	2	4/0.25(S)	4/1(I)	64/0.25(I)	64/1(I)
109149	>64	1	1	0.5	2	16/0.25(S)	64/2(I)	64/1(I)	1/1(I)
109152	>64	1	1	0.5	1	1/0.25(S)	64/1(I)	2/1(I)	0.5/1(I)
NPRC 3.8.656	>64	1	1	0.25	1	2/0.25(S)	64/2(I)	64/0.5(I)	64/2(I)
NPRC 3.8.655	>64	1	2	0.5	2	4/0.25(S)	64/4(I)	16/0.25(I)	64/4(I)
NPRC 3.8.654	>64	1	1	0.5	2	16/0.25(S)	64/2(I)	64/1(I)	64/4(I)
NPRC 3.8.653	>64	1	1	0.5	2	16/0.25(S)	64/2(I)	64/1(I)	1/1(I)
NPRC 3.8.652	>64	0.5	1	1	2	2/0.125(S)	64/2(I)	16/0.25(S)	64/4(I)
Δ*ABC2*	>64	1	1	0.25	1	2/0.5(I)	4/0.5(I)	64/0.5(I)	64/2(I)
*E. alcalophila*									
CBS00017	>64	0.5	0.5	0.0625	0.25	1/0.125(S)	16/0.125(S)	64/0.125(I)	16/0.0625(S)
CBS00038	>64	4	8	4	2	64/8(I)	64/16(I)	64/8(I)	64/4(I)
CBS00045	>64	1	1	0.25	1	2/0.25(S)	16/0.25(S)	64/0.5(I)	64/2(I)
CBS00046	>64	0.25	0.5	1	0.5	1/0.25(I)	2/0.5(I)	2/1(I)	2/0.5(I)
CBS273.37	>64	0.5	0.5	0.25	0.5	64/1(I)	1/0.5(I)	4/0.5(I)	64/1(I)
CBS286.47	>64	0.5	1	2	1	1/0.5(I)	0.25/1(I)	8/2(I)	64/2(I)
CBS840.69	>64	16	16	16	4	64/32(I)	64/32(I)	64/32(I)	64/8(I)
*F. pedrosoi*									
FP001	>64	0.5	2	0.5	0.5	64/0.125(I)	64/4(I)	64/1(I)	16/1(I)
07633	>64	2	4	2	2	64/4(I)	64/8(I)	64/1(I)	2/4(I)
07631	>64	0.0625	1	0.5	0.25	64/0.125(I)	2/0.5(I)	1/0.25(I)	2/0.25(I)
*F. monophora*									
Fm001	>64	0.0625	2	0.5	1	2/0.03125(I)	1/1(I)	2/0.25(I)	1/0.5(I)
*E. verrucosa*	>64								
Ev001	>64	0.0625	0.25	0.25	0.25	64/0.125(I)	64/0.5(I)	64/0.5(I)	64/0.5(I)
*A. niger*									
AN1	>64	1	1	1	1	4/0.0625(S)	8/0.5(I)	32/1(I)	32/1(I)
AN2	>64	1	0.5	0.5	1	4/0.03125(S)	32/0.5(I)	32/0.5(I)	32/1(I)
AN3	>64	1	1	1	0.5	4/0.03125(S)	32/1(I)	32/1(I)	32/0.5(I)
AN4	>64	1	1	0.5	1	8/0.03125(S)	32/1(I)	32/0.5(I)	32/1(I)
AN5	>64	1	1	0.5	1	2/0.03125(S)	32/1(I)	32/0.5(I)	32/1(I)
AN6	>64	1	1	1	2	16/0.03125(S)	32/1(I)	32/1(I)	32/2(I)
AN7	>64	0.125	1	0.25	0.5	2/0.03125(S)	1/0.5(I)	32/0.25(I)	32/0.5(I)
Quality control									
ATCC 204304	>64	0.5	1	0.5	0.25	8/0.5(I)	32/1(I)	16/0.25(I)	8/0.5(I)
ATCC 22019	>64	0.125	0.5	0.125	0.25	32/0.125(I)	16/0.25(I)	16/0.125(I)	32/0.25(I)

MICs were the concentrations that achieved 100% growth inhibition. For FICI calculations, the concentration of 128 μg/mL were used when MICs were >64 μg/mL.

ITC, itraconazole; VOR, voriconazole; POS, posaconazole; ISA, isavuconazole; DTX, docetaxel; S, synergy (FICI ≤ 0.5); I, indifference (FICI from >0.5 to ≤4); FICI: fractional inhibitory concentration index.

Comprehensive analysis of synergistic data across all 43 strains showed that the DTX + POS combination exhibited the optimal synergistic effect: the synergy rate reached 100% for *A. niger* and 87.5% for *E. dermatitidis* (Figure 2; Table 2).

**Table 2:** Summary of drug interaction for the combination of DTX and azoles.

Species (*n*)	*n* (%) of strains showing synergism or antagonism for the combination							
	BA+POS		BA+ITC		BA+VOR		BA+ISA	
	S	I	S	I	S	I	S	I
*E. dermatitidis* (24)	21 (87.5%)	3 (12.5%)	5 (20.8%)	19 (79.2%)	3 (12.5%)	21 (87.5%)	0 (0.0%)	24 (100.0%)
*E. alcalophila* (7)	2 (28.6%)	5 (71.4%)	2 (28.6%)	5 (71.4%)	0 (0.0%)	7 (100.0%)	1 (14.3%)	6 (85.7%)
*F. pedrosoi* (3)	0 (0.0%)	3 (100.0%)	0 (0.0%)	3 (100.0%)	0 (0.0%)	3 (100.0%)	0 (0.0%)	3 (100.0%)
*F. monophora* (1)	0 (0.0%)	1 (100.0%)	0 (0.0%)	1 (100.0%)	0 (0.0%)	1 (100.0%)	0 (0.0%)	1 (100.0%)
*E. verrucosa* (1)	0 (0.0%)	1 (100.0%)	0 (0.0%)	1 (100.0%)	0 (0.0%)	1 (100.0%)	0 (0.0%)	1 (100.0%)
*A. niger* (7)	7 (100.0%)	0 (0.0%)	0 (0.0%)	7 (100.0%)	0 (0.0%)	7 (100.0%)	0 (0.0%)	7 (100.0%)
Total (43)	30 (69.8%)	13 (30.2%)	7 (16.3%)	36 (83.7%)	3 (7.0%)	40 (93.0%)	1 (2.3%)	42 (97.7%)

ITR, itraconazole; VOR, voriconazole; POS, posaconazole; DTX, docetaxel; S, synergy (FICI ≤ 0.5); I, indifference (no interaction, FICI from >0.5 to ≤4).

For *E. dermatitidis*, the overall POS MIC ranged from 0.25 to 1 μg/mL. Strains with synergistic effects showed MIC values of 0.25–1 μg/mL, while all non-synergistic strains had an MIC of 1 μg/mL. For *E. alcalophila*, the total MIC distribution was 0.25–16 μg/mL. Synergism was detected only in strains with an MIC of 0.5 μg/mL, and no synergistic activity was found in strains with MIC values from 0.25 to 16 μg/mL. No synergistic effect was observed in all tested *F. pedrosoi* isolates, with POS MIC values ranging from 0.0325 to 2 μg/mL.

Strains with higher baseline MICs did not exhibit enhanced synergism, and strains with different azole susceptibility phenotypes presented similar responses to combination treatment. These findings indicate that the synergistic activity of the DTX + POS combination is largely independent of the baseline azole susceptibility of fungal strains.

### 3.2 DTX-mediated rhodamine efflux

**Figure 3:**
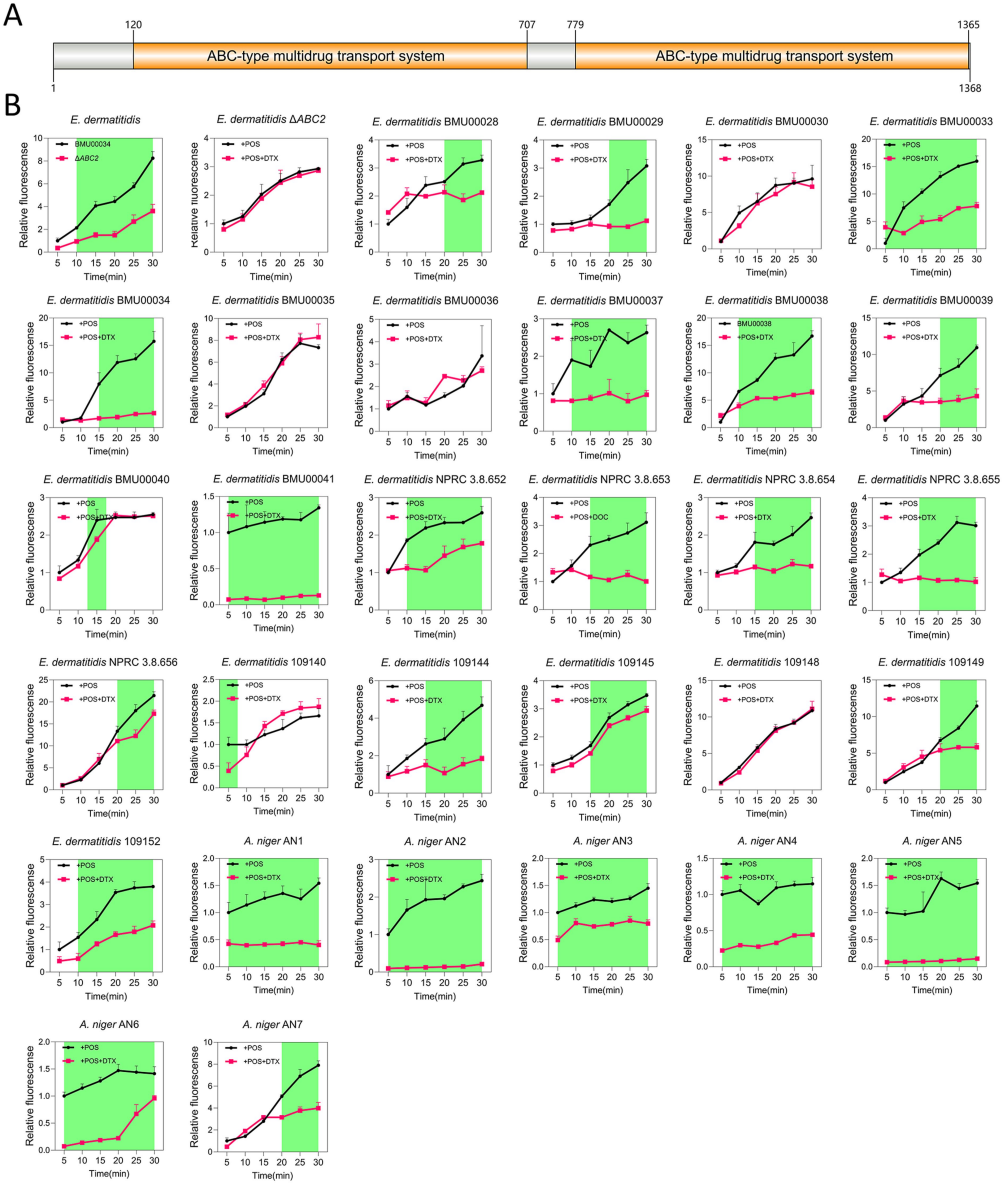
POS combined with DTX reduces efflux pump activity in dematiaceous fungi. **(A)** Schematic diagram of the amino acid domain structure of ABC2. **(B)** POS combined with DTX reduces efflux pump activity in target strains (*E. dermatitidis* and *A. niger*). For data processing: The fluorescence intensity of the POS monotherapy group at 5 min was set as the baseline for normalization of all fluorescence signals. Relative fluorescence intensity was defined as the ratio of fluorescence intensity at each timepoint to the baseline fluorescence value of the POS-only group at 5 min. Statistical analysis was performed by Two-way ANOVA with Tukey’s test for multiple comparisons. The green shaded area indicates statistically significant differences (*p* < 0.05).

ABC transporters and the Major Facilitator Superfamily (MFS) together constitute a large, diverse group of secondary transporters, including uniporters, symporters and antiporters, which are closely associated with fungal drug resistance. Notably, the Δ*ABC2* strain, which showed no synergistic effect, is a gene-deficient mutant derived from the *E. dermatitidis* BMU00034 strain (which exhibited synergy) (Yuan et al., 2025). The deleted gene encodes a protein containing two ABC domains, suggesting it may be associated with drug efflux pumps (Figure 3A). Consistent with this, our results confirmed that efflux pump activity was significantly reduced in the Δ*ABC2*. Notably, the DTX + POS combination produced remarkable synergistic antifungal activity in BMU00034 strains. In contrast, this synergistic effect was completely abolished in Δ*ABC2*, where the drug combination only exhibited an indifferent interaction. In addition, efflux pump activity was significantly attenuated in the Δ*ABC2*. The above phenotypic differences further confirm that the ABC transporter encoded by *ABC2* may contribute to the synergistic effect. The synergistic efficacy of DTX + POS combination is likely mediated by altered efflux pump activity. We hypothesize that the synergistic effect generated by co-treatment with POS and DTX is associated with changes in efflux pump activity. To test this hypothesis, we examined efflux pump activity in the fungal species with the most prominent synergistic effects (*E. dermatitidis* and *A. niger*) in the presence of DTX. The results showed that, compared to cultures containing POS alone, efflux pump activity was significantly reduced when DTX was added. Among the 31 fungal strains tested, 26 (83.9%) exhibited a significant decrease in efflux pump activity, with no observed increase in activity. Notably, *E. dermatitidis* BMU00030 and 109140, which showed no synergistic effect, provided further support: efflux pump activity in strain BMU00030 remained unchanged after DTX addition, while in strain 109140, only a transient decrease in efflux pump activity was detected at the 5-min time point (Figure 3B).

### 3.3 Modulation of efflux pump gene transcription by DTX+POS combination

Canonical efflux pump genes (*mdr1*, *abcA*, *abcB*, *mdr2*, *atrF*) known to modulate azole susceptibility in pathogenic fungi were used as references for sequence homology alignment in the NCBI database (Supplementary Table S3) (Czajka et al., 2023; Fraczek et al., 2013; Ibe et al., 2024; Li et al., 2023; Paul et al., 2013). Homologous coding genes with the highest structural similarity were screened for transcriptional quantification. All proteins encoded by these candidate genes contain conserved transporter domains, implying their conserved function in antifungal drug extrusion (Supplementary Figure S1).

All tested *E. dermatitidis* strains were divided into two groups according to whether the DTX + POS combination exerted synergistic antifungal effects: non-synergistic strains BMU00030 (FICI = 0.563) and 109140 (FICI = 2.500), and strains with prominent synergistic activity including BMU00036 (FICI = 0.266), 109152 (FICI = 0.258) and BMU00040 (FICI = 0.266). Under single POS treatment, the transcriptional level of the ABC transporter gene *ABC1* showed no significant difference between the non-synergistic strains BMU00030 and 109140. In contrast, the expression of *ABCB1* was markedly higher in synergistic strains BMU00036, 109152 and BMU00040 compared with the two non-synergistic strains.

**Figure 4:**
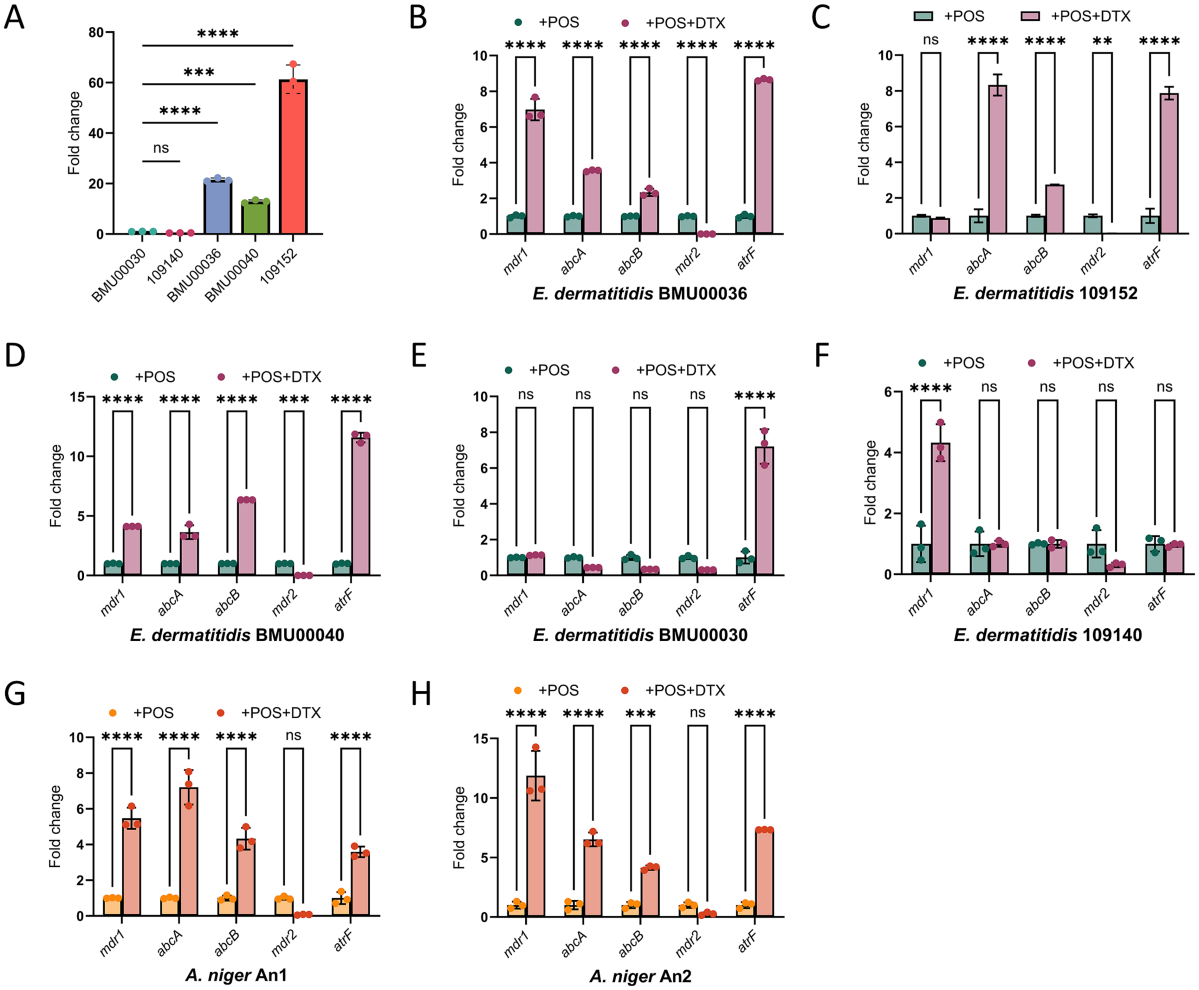
RT-qPCR analysis of efflux pump gene expression in *E. dermatitidis* and *A. niger.* **(A)** Fold changes of *ABC2* in different *E. dermatitidis*. **(B–F)** Relative expression of efflux genes in *E. dermatitidis* strains. **(G,H)** Relative expression of efflux genes in *A. niger* strains. Statistical analyses were performed by Two-way ANOVA with Tukey’s test for multiple comparisons. ns, *p* > 0.05; **p < 0.01; ***p < 0.001; ****p < 0.0001. POS, posaconazole; DTX, docetaxel.

Following co-treatment with DTX + POS combination, obvious transcriptional alterations of ABC-family transporters were observed in synergistic strains BMU00036, 109152 and BMU00040: the transcript levels of *abcA* and *abcB* were significantly upregulated, while *mdr2* was notably downregulated. By contrast, no significant transcriptional changes of these three genes were detected in non-synergistic strains BMU00030 and 109140. Distinct expression patterns were observed for *atrF* and *mdr1* between groups. Among non-synergistic strains, only *atrF* was upregulated in strain BMU00030, whereas only *mdr1* was upregulated in strain 109140. For synergistic strains BMU00036 and BMU00040, both *atrF* and *mdr1* were synchronously and significantly overexpressed (Figure 4).

In addition, obvious transcriptional shifts of efflux pump genes were also detected in *A. niger*. In An1 and An2, *mdr1*, *abcA* and *atrF* were significantly upregulated, *abcB* was downregulated, and no obvious expression change was observed for *mdr2* (Figure 4).

## 4 Discussion

**Figure 5:**
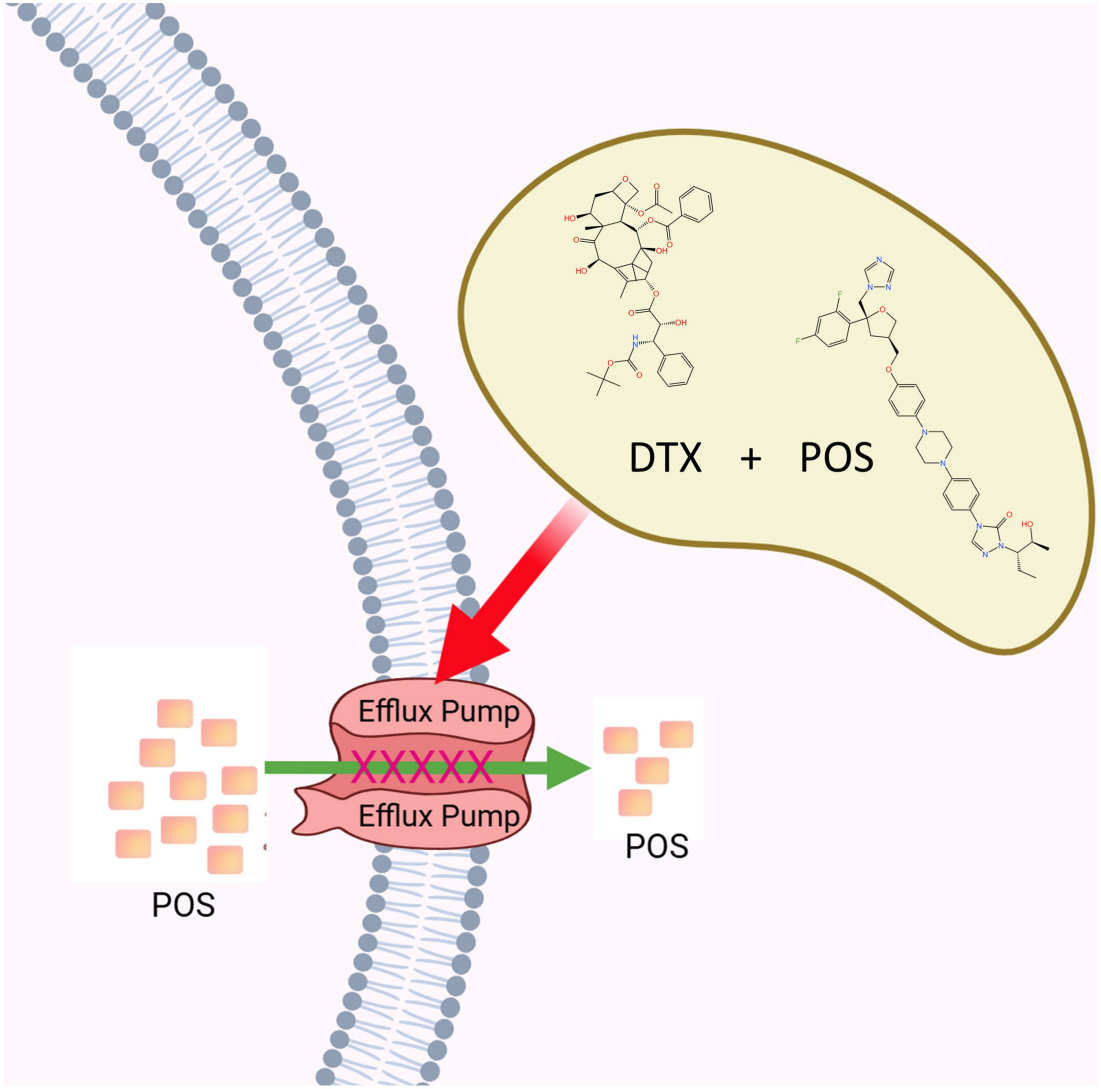
The DTX + POS combination might potentially modulate efflux pump activity. Note: Schematic diagram of the synergistic antifungal mechanism of DTX + POS combination. DTX may suppress the transport function of fungal efflux pumps, potentially reducing the extracellular efflux of POS and elevating its intracellular accumulation, which consequently contributes to synergistic antifungal effects. POS, posaconazole; DTX, docetaxel.

To tackle the escalating challenge of antifungal resistance in pathogenic fungi, combination therapy has emerged as a strategic therapeutic approach. This study confirmed that the DTX + POS combination exerts prominent synergistic effects against *E. dermatitidis* and *A. niger*. Results of efflux pump activity assays demonstrated that compared with POS monotherapy, the efflux pump activity of fungi was markedly reduced after co-treatment with DTX, accompanied by significant alterations in the expression levels of transporter genes. These findings indicate that the generation of synergistic antifungal effects is correlated with efflux pump activity (Figure 5).

Tubulin has been reported to serve as a promising therapeutic target for both anticancer and antifungal treatments (Li et al., 2025). DTX is a semi-synthetic taxane anticancer agent characterized by high efficacy and a broad spectrum of activity. It exhibits significant therapeutic effects against various malignant tumors, including breast cancer, non-small cell lung cancer, and PCa. Its core mechanism of action involves binding to specific sites on tubulin (primarily *β*-tubulin), thereby inhibiting microtubule depolymerization and stabilizing microtubule structures (Kathawala et al., 2014; Whelan, 2002). The core constituent proteins of microtubules are generally highly conserved across eukaryotes. As an essential biomacromolecule within eukaryotic cells, it plays a critical role in maintaining cellular morphology, facilitating cell division and sustaining cell viability (Guimar Es et al., 2021). Similarly, DTX may engage in the regulation of fungal physiological metabolism; it might interfere with intracellular cytoskeleton-based cargo transport and vesicle trafficking, which in turn indirectly modulates the activity of membrane transporters and fungal susceptibility to azole antifungals.

DTX binds to free tubulin in the cytoplasm, and an adequate intracellular concentration is vital to stabilize the microtubules (Thadani-Mulero et al., 2012). The intracellular concentration of DTX depends on the ratio of drug influx and efflux pumps (Sanchez et al., 2009). Therefore, downregulation of influx transporter activity or upregulation of efflux transporters activity may play crucial roles in DTX efficacy (de Morree et al., 2016; Sanchez et al., 2011).

In *C. albicans* and *A. fumigatus*, the efflux pumps involved in multidrug resistance mainly belong to the ABC superfamily (represented by Cdr1) and the MFS (represented by Mdr1), which transport drugs powered by ATP hydrolysis and proton concentration gradientsrespectively (Banerjee et al., 2021; Costa et al., 2014; Czajka et al., 2023). Multiple studies have shown that overexpression of efflux pumps alone is sufficient to confer azole resistance, and efflux pump detection may serve as one of the important methods for assessing fungal resistance (Bhattacharya et al., 2016; Sanguinetti et al., 2005).

ABCB1 demonstrates high-affinity binding to DTX and can efficiently pump DTX out of treated tumor cells hence decreasing the efficacy of stabilizing microtubules (Kroon et al., 2016; Martin et al., 2015). The drug resistance that frequently emerges during DTX treatment is also attributed to changes in the activity of ABCB1. This suggests that DTX likely interacts with efflux pump-related proteins by competing for their substrate-binding sites, rather than activating them. By the same inference, in the POS and DTX combination regimen, some efflux pumps may transport DTX preferentially, thereby potentially reducing the efflux of POS. This leads to elevated intracellular POS concentrations and ultimately produces the synergistic antifungal effect.

Notably, POS shows the highest synergy rate, while the synergy rates of VOR, ITC, and ISA are relatively low. This may be attributed to the structure of POS, which contains a tetrahydrofuran ring and a long-chain alkyl side chain. This structural feature significantly enhances the lipophilicity and tissue penetration of POS, particularly its ability to penetrate fungal biofilms (Alsulaimany et al., 2025; Sagatova et al., 2016; Seyedmousavi et al., 2014; Xiao et al., 2025). Such a highly permeable structure may competitively bind to the binding sites of efflux pumps. DTX may competitively bind to the substrate-binding pocket of fungal ABC transporters (homologous to P-gp in cancer cells), thereby inhibiting POS efflux. As a substrate of efflux pumps, DTX can competitively bind to efflux pumps with POS, thereby reducing both the energy required for POS efflux and the availability of binding sites. The high permeability of POS results in less efflux compared with VOR, ITC, and ISA, and this feature more substantially reduces the ‘clearance’ efficiency of efflux pumps for POS, manifested as a functional reduction in efflux pump activity and a marked synergistic effect. In contrast to *A. niger*, the synergistic rate did not reach 100% in *E. dermatitidis*. This phenomenon may be attributed to substantial inter-strain variations in susceptibility to the DTX + POS combination within the same species, which are collectively driven by multiple intrinsic strain-specific factors. Basal transcription levels of efflux pump-related genes (such as *ABC1*) differ across strains, leading to distinct baseline efflux capacities. Strains with higher intrinsic efflux activity tend to exhibit prominent synergistic antifungal effects mediated by DTX, while the absence of synergism in certain strains may correlate with low basal expression of efflux pumps. In addition, single-nucleotide polymorphisms or minor mutations within drug target genes and efflux pump regulatory genes can alter the overall drug tolerance of individual strains. Variations in cell membrane composition, cell wall architecture and melanin content among strains influence drug penetration efficiency and intracellular drug accumulation. Notably, melanin, a unique component of the cell wall in dematiaceous fungi, can physically adsorb antifungal agents to further reduce the effective intracellular drug concentration, ultimately resulting in inconsistent combinatorial efficacy across strains.

As a classic chemotherapeutic agent, DTX generally suffers from poor water solubility and insufficient bioavailability; additionally, its severe toxic side effects greatly restrict the clinical dosage and therapeutic efficacy (Jurczyk et al., 2022). When administered at the standard anti-tumor dose of 100 mg/m^2^, the incidence of neutropenia in patients reaches 50–80%. Even with premedication using corticosteroids, clinical doses are frequently reduced to 30–75 mg/m^2^ to manage toxicities (Baker et al., 2006; Lyseng-Williamson et al., 2005). At present, a variety of novel delivery carriers including liposomes, solid lipid nanoparticles, dendrimers, nanoparticles and micelles have been successively developed, and several nanoformulations have advanced into clinical trials (Jurczyk et al., 2022). Novel co-delivery carriers are also under development. For instance, ligand-targeted nanoparticles (NPs) are exploited for the co-delivery of DTX with other anti-cancer agents. As a representative example, NPs co-loaded with DTX and gambogic acid (Gba) have yielded promising clinical trial outcomes for targeting prostate-specific membrane antigen (PSMA)-positive cancer cells (Afsharzadeh et al., 2020; Jurczyk et al., 2022). Meanwhile, pH-sensitive NPs co-delivering DTX and dihydroartemisinin (Dha) have been investigated for the treatment of triple-negative breast cancer (Tao et al., 2021). Such technologies can optimize the physicochemical properties of drugs, achieve targeted delivery, and effectively reduce the systemic toxicity of DTX. Infections caused by dematiaceous fungi and *A. niger* are mostly localized to the skin, subcutaneous tissues, and mucosa, which are highly suitable for local intervention. In this study, a low concentration of DTX (2 μg/mL) exerted significant synergistic antifungal effects with POS and efficiently inhibited *E. dermatitidis* and *A. niger in vitro*. Future studies can focus on the development of topical formulations for clinical application.

This study reveals that DTX can synergistically enhance the antifungal activity of POS against dematiaceous fungi. These findings offer new strategies for optimizing antifungal combination therapy, deepen our understanding of fungal resistance mechanisms, and propose innovative approaches for the clinical management of fungal infections. However, the current research evidence is still limited to *in vitro* models. While R6G efflux assays support reduced efflux activity, future studies should validate this mechanism via Western blotting. Future *in vivo* studies may employ immunosuppressed murine models of *E. dermatitidis* cutaneous infection to the DTX + POS combination synergy observed *in vitro*, with outcome measures including lesion size and fungal burden in tissues.

Nevertheless, the present study has several major limitations, as all experiments were performed exclusively *in vitro*. On the one hand, we only inferred altered fungal efflux activity based on rhodamine 6G efflux assays and transcriptional changes of partial efflux pump genes. Western blot analysis was not carried out to validate the underlying mechanisms at both transcriptional and protein levels. Moreover, intracellular drug accumulation measurement and molecular docking simulation were not implemented, which prevents us from clarifying the direct molecular interaction between the drugs and efflux transporters. On the other hand, time-kill curve tests and biofilm assays were not included in this study; thus, the fungicidal kinetics and anti-biofilm activity of the drug combination remain uncharacterized. In addition, mammalian cytotoxicity evaluation was absent, and no immunosuppressed mouse model of cutaneous *E. dermatitidis* infection was established for *in vivo* efficacy verification. Accordingly, the safety and *in vivo* therapeutic potency of this combinatorial regimen require further systematic investigation.

It should be noted that a total of 43 fungal strains were enrolled in this study, predominantly *E. dermatitidis* alongside a small number of *A. niger* strains. Hence, the conclusions regarding synergistic antifungal effects and associated mechanisms are only applicable to these two species and cannot be generalized to all dematiaceous fungi. Beyond the dematiaceous fungi and *A. niger* tested in the present study, this DTX + POS combination holds promising therapeutic potential against other prevalent clinical pathogenic fungi. *A. fumigatus* and *C. auris* are two clinically prevalent pathogens with severe azole resistance issues: azole resistance in *A. fumigatus* is primarily mediated by overexpression of ABC transporters AbcA, AbcB and Mdr1, while *C. auris* frequently develops pan-azole resistance via upregulation of efflux pumps (Czajka et al., 2023; Fraczek et al., 2013; Ibe et al., 2024; Li et al., 2023; Paul et al., 2013). Considering DTX’s potential to inhibit fungal efflux pumps, we speculate that the DTX-POS combination could similarly inhibit *A. fumigatus* and *C. auris*, a hypothesis requiring rigorous *in vitro* and *in vivo* confirmation. Future research will expand the strain library by including dematiaceous fungi covering additional genera and species to verify the generalizability of our present findings. Meanwhile, the unperformed *in vitro* functional assays, cytotoxicity evaluations and *in vivo* animal efficacy studies described above will be supplemented.

In summary, DTX synergistically potentiates the antifungal activity of POS against *E. dermatitidis* and *A. niger*. These findings not only offer a novel perspective for developing optimized combinatorial antifungal regimens, but also deepen our understanding of fungal drug resistance mechanisms, paving a new path for the clinical management of invasive fungal infections.

## 5 Conclusion

DTX in combination with POS exerts significant synergistic antifungal effects against *E. dermatitidis* and *A. niger*, markedly reducing POS’s minimum inhibitory concentration, and thus provides a novel potential strategy for treating clinically challenging infections caused by these fungi.

## Data Availability

The datasets presented in this study can be found in online repositories. The names of the repository/repositories and accession number(s) can be found at: https://www.ncbi.nlm.nih.gov/, PP069948-PP070390.

## Footnotes

1
https://blast.ncbi.nlm.nih.gov/Blast.cgi
