# Supplementary material for: In vitro study on synergistic antifungal effects of docetaxel combined with azoles against dematiaceous fungi and Aspergillus niger
Source: Front Microbiol. 2026 Jul 17;17:1832946. doi: 10.3389/fmicb.2026.1832946 (PMC13423856; doi:10.3389/fmicb.2026.1832946)
Supplement: Supplementary file 1 [file Table_1.DOCX]

###### TABLE S1 Source of tested strains

| Strains | Isolation site | Underlying disease |
| --- | --- | --- |
| *E. dermatitidis* |  |  |
| BMU00028 | Brain | Phaeohyphomycosis |
| BMU00029 | Skin | Phaeohyphomycosis |
| BMU00030 | Skin | Phaeohyphomycosis |
| BMU00031 | Skin | Phaeohyphomycosis |
| BMU00034 | Skin | Phaeohyphomycosis |
| BMU00035 | Skin | Phaeohyphomycosis |
| BMU00036 | Skin | Phaeohyphomycosis |
| BMU00037 | Skin | Phaeohyphomycosis |
| BMU00038 | Skin | Phaeohyphomycosis |
| BMU00039 | Skin | Phaeohyphomycosis |
| BMU00040 | Skin | Phaeohyphomycosis |
| BMU00041 | Skin | Phaeohyphomycosis |
| 109140 | Skin | Phaeohyphomycosis |
| 109144 | Skin | Phaeohyphomycosis |
| 109145 | Skin | Phaeohyphomycosis |
| 109148 | Skin | Phaeohyphomycosis |
| 109149 | Skin | Phaeohyphomycosis |
| 109152 | Skin | Phaeohyphomycosis |
| NPRC 3.8.656 | Skin | Phaeohyphomycosis |
| NPRC 3.8.655 | Skin | Phaeohyphomycosis |
| NPRC 3.8.654 | Skin | Phaeohyphomycosis |
| NPRC 3.8.653 | Skin | Phaeohyphomycosis |
| NPRC 3.8.652 | Skin | Phaeohyphomycosis |
| Δ*ABC2* | Laboratory constructed | Not applicable |
| *E. alcalophila* |  |  |
| CBS00017 | Skin | Phaeohyphomycosis |
| CBS00038 | Skin | Phaeohyphomycosis |
| CBS00045 | Skin | Phaeohyphomycosis |
| CBS00046 | Skin | Phaeohyphomycosis |
| CBS273.37 | Skin | Phaeohyphomycosis |
| CBS286.47 | Skin | Phaeohyphomycosis |
| CBS840.69 | Skin | Phaeohyphomycosis |
| *F. pedrosoi* |  |  |
| FP001 | Skin | Phaeohyphomycosis |
| 07633 | Skin | Phaeohyphomycosis |
| 07631 | Skin | Phaeohyphomycosis |
| *F. monophora* |  |  |
| Fm001 | Skin | Phaeohyphomycosis |
| *E. verrucosa* |  |  |
| Ev001 | Skin | Phaeohyphomycosis |
| *A. niger* |  |  |
| AN1 | External auditory canal | Otomycosis |
| AN2 | External auditory canal | Otomycosis |
| AN3 | External auditory canal | Otomycosis |
| AN4 | External auditory canal | Otomycosis |
| AN5 | External auditory canal | Otomycosis |
| AN6 | External auditory canal | Otomycosis |
| AN7 | External auditory canal | Otomycosis |

**TABLE S2 Primer sets and corresponding amplification targets**

| Target gene | Primer | Primer DNA sequence (5’-3’) |
| --- | --- | --- |
| ITS | ITS1 | TCCGTAGGTGAACCTGCGG |
|  | ITS4 | TCCTCCGCTTATTGATATGC |
| calmodulin | cmd5 | CCGAGTACAAGGAGGCCTTC |
|  | cmd6 | CCGATAGAGGTCATAACGTGG |
| beta-tubulin | Bt2a | GGTAACCAAATCGGTGCTGCTTTC |
|  | Bt2b | ACCCTCAGTGTAGTGACCCTTGGC |

###### TABLE S3 Primers used for RT-qPCR

| Species | Target gene | Primer | Primer DNA sequence (5’–3’) |
| --- | --- | --- | --- |
| *E. dermatitidis* | *ABC2* | EdABC2-F | CACATGGTGAGACGAGGTATTC |
|  |  | EdABC2-F | AGAAAGCCCACGCACATAA |
|  | *mdr1* | Edmdr1-F | CTTCGTTCTGGGATACGCAATA |
|  |  | Edmdr1-R | GTGCCAATGTCCCTATGTAGAC |
|  | *abcA* | EdabcA-F | CTCCCGCTGCTATTCTCATATT |
|  |  | EdabcA-R | GTATGCGACAGGGTCAAGATAG |
|  | *abcB* | EdabcB-F | GGTGCTAGGCGACGAATAAA |
|  |  | EdabcB-R | GCATCTTTGCAGGACGTTATTT |
|  | *mdr2* | Edmdr2-F | TCGGCTGTCCACTATCAAAC |
|  |  | Edmdr2-R | GGTCGACTACTGAGGTCTCTAT |
|  | *atrF* | EdatrF-F | GAGCTTACGAGGATGAGCTTAC |
|  |  | EdatrF-R | CATAGATGAGCGAGTCCTTGAC |
|  | *actin* | Edactin-F | TGCTGGAGCAAGGGTAATAAG |
|  |  | Edactin-R | GATGTGACGAGAGACAGAAACA |
| *A. niger* | *mdr1* | Anmdr1-F | GATTGTCGGGAGTGGATTCTT |
|  |  | Anmdr1-R | GAGAACACTAGCCGCATACTC |
|  | *abcA* | AnabcA-F | CAAGATCACCCTACACGCTATC |
|  |  | AnabcA-R | CGGCCAAGGTCATACTCATATC |
|  | *abcB* | AnabcB-F | GGTGGACAACACCCTACATATT |
|  |  | AnabcB-R | CAACAGAACAACGCCCAAAG |
|  | *mdr2* | Anmdr1-F | TTGAGCGTACCGATGTTCTATG |
|  |  | Anmdr1-R | CTCTCCAACGATACGGAGAATG |
|  | *atrF* | AnatrF-F | CATCATTTGGTGTCCAGCTTTATG |
|  |  | AnatrF-R | GCGGACAGAACACAGAGAATAG |
|  | *actin* | Anactin-F | ACCCTCAGATACCCCATTGA |
|  |  | Anactin-R | CTGGGTCATCTTCTCACGG |


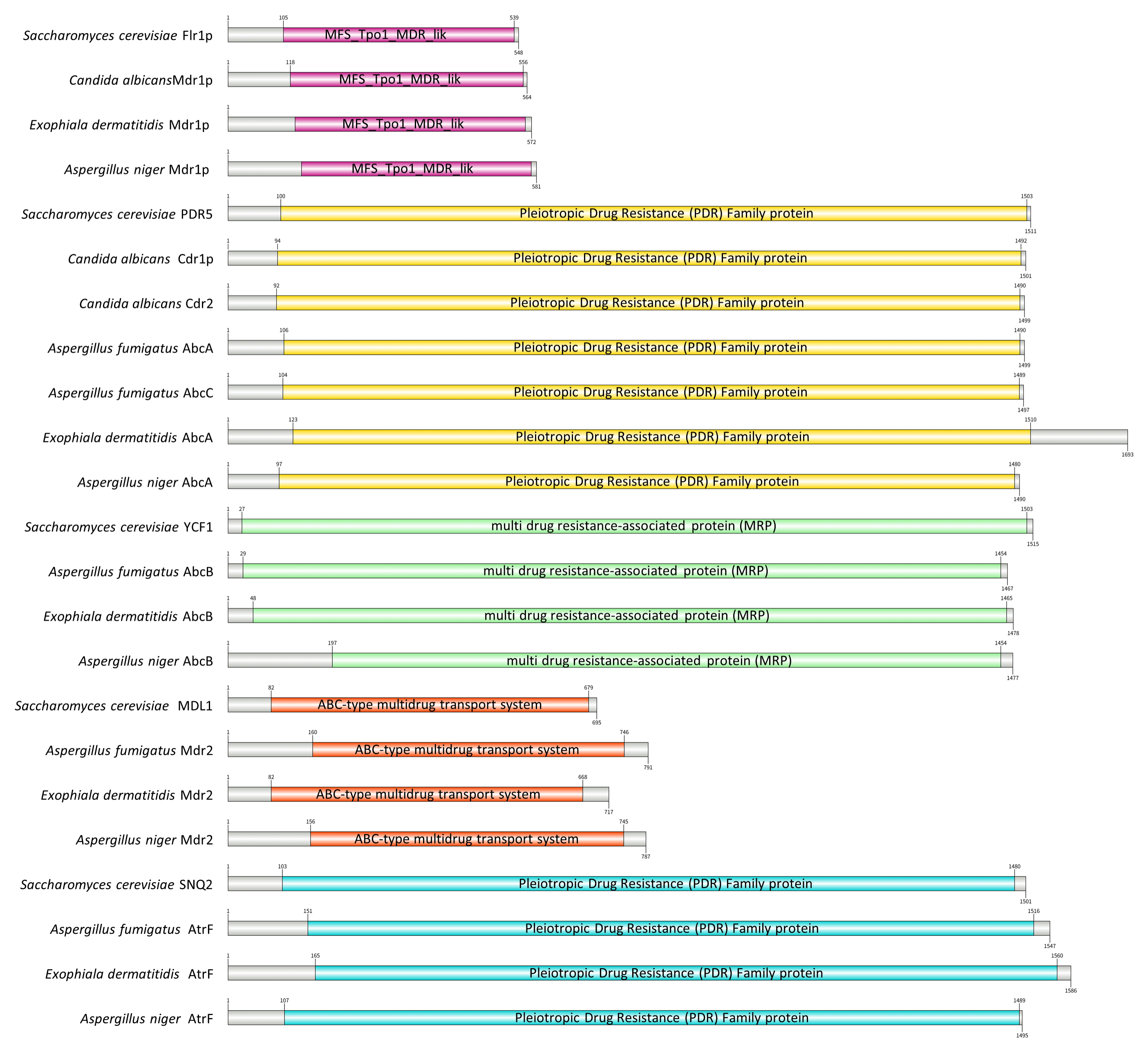


**FIGURE 1 Schematic diagram of conserved domain alignment of efflux transporters**

**Note:** *Saccharomyces cerevisiae* Flr1p, GenPept accession no. NP_009562.1; *Candida albicans* Mdr1p, GenPept accession no. XP_719165.2; *Exophiala dermatitidis* Mdr1p, GenPept accession no. XP_009154539.1; *Aspergillus niger* Mdr1p, GenPept accession no. XP_001398631.1; *Saccharomyces cerevisiae* PDR5, GenPept accession no. NP_014796.3; *Candida albicans* Cdr1p, GenPept accession no. XP_723209.2; *Candida albicans* Cdr2, GenPept accession no. XP_723169.1; *Aspergillus fumigatus* AbcA, GenPept accession no. XP_755847.1; *Aspergillus fumigatus* AbcC, GenPept accession no. XP_752803.1; *Exophiala dermatitidis* AbcA, GenPept accession no. XP_009153950.1; *Aspergillus niger* AbcA, GenPept accession no. XP_001396831.1; *Saccharomyces cerevisiae* YCF1, GenPept accession no. NP_010419.3; *Aspergillus fumigatus* AbcB, GenPept accession no. XP_077660068.1; *Exophiala dermatitidis* AbcB, GenPept accession no. XP_009157190.1; *Aspergillus niger* AbcB, GenPept accession no. XP_059605411.1; *Saccharomyces cerevisiae* MDL1, GenPept accession no. NP_013289.1; *Aspergillus fumigatus* Mdr2, GenPept accession no. XP_751826.1; *Exophiala dermatitidis* Mdr2, GenPept accession no. XP_009158370.1; *Aspergillus niger* Mdr2, GenPept accession no. XP_001402067.1; *Saccharomyces cerevisiae* SNQ2, GenPept accession no. NP_010294.1; *Aspergillus fumigatus* AtrF, GenPept accession no. XP_747642.1; *Exophiala dermatitidis* AtrF, GenPept accession no. XP_009153951.1; *Aspergillus niger* AtrF, GenPept accession no. XP_001390738.1; The figure was generated using the online tool IBS 2.0 ([https://ibs.renlab.org/](https://ibs.renlab.org/)).
